# Melatonin protects cardiac microvasculature against ischemia/reperfusion injury via suppression of mitochondrial fission‐VDAC1‐HK2‐mPTP‐mitophagy axis

**DOI:** 10.1111/jpi.12413

**Published:** 2017-04-27

**Authors:** Hao Zhou, Ying Zhang, Shunying Hu, Chen Shi, Pingjun Zhu, Qiang Ma, Qinhua Jin, Feng Cao, Feng Tian, Yundai Chen

**Affiliations:** ^1^ Department of Cardiology Chinese PLA General Hospital Beijing China; ^2^ Key Laboratory of Carcinogenesis and Translational Research (Ministry of Education/Beijing) Department of Radiation Oncology Peking University Cancer Hospital and Institute Beijing China

**Keywords:** AMP‐activated protein kinase α, cardiac microcirculation endothelial cells, hexokinase 22, melatonin, microvascular IR injury, mitochondrial fission, mitochondrial permeability transition pore opening, voltage‐dependent anion channel 1

## Abstract

The cardiac microvascular system, which is primarily composed of monolayer endothelial cells, is the site of blood supply and nutrient exchange to cardiomyocytes. However, microvascular ischemia/reperfusion injury (IRI) following percutaneous coronary intervention is a woefully neglected topic, and few strategies are available to reverse such pathologies. Here, we studied the effects of melatonin on microcirculation IRI and elucidated the underlying mechanism. Melatonin markedly reduced infarcted area, improved cardiac function, restored blood flow, and lower microcirculation perfusion defects. Histological analysis showed that cardiac microcirculation endothelial cells (CMEC) in melatonin‐treated mice had an unbroken endothelial barrier, increased endothelial nitric oxide synthase expression, unobstructed lumen, reduced inflammatory cell infiltration, and less endothelial damage. In contrast, AMP‐activated protein kinase α (AMPKα) deficiency abolished the beneficial effects of melatonin on microvasculature. In vitro, IRI activated dynamin‐related protein 1 (Drp1)‐dependent mitochondrial fission, which subsequently induced voltage‐dependent anion channel 1 (VDAC1) oligomerization, hexokinase 2 (HK2) liberation, mitochondrial permeability transition pore (mPTP) opening, PINK1/Parkin upregulation, and ultimately mitophagy‐mediated CMEC death. However, melatonin strengthened CMEC survival via activation of AMPKα, followed by p‐Drp1^S616^ downregulation and p‐Drp1^S37^ upregulation, which blunted Drp1‐dependent mitochondrial fission. Suppression of mitochondrial fission by melatonin recovered VDAC1‐HK2 interaction that prevented mPTP opening and PINK1/Parkin activation, eventually blocking mitophagy‐mediated cellular death. In summary, this study confirmed that melatonin protects cardiac microvasculature against IRI. The underlying mechanism may be attributed to the inhibitory effects of melatonin on mitochondrial fission‐VDAC1‐HK2‐mPTP‐mitophagy axis via activation of AMPKα.

## 1 Introduction

Despite the successful revascularization of occluded epicardial vessels, the postischemia failure of reperfusion to the microvascular bed termed as microcirculatory ischemia/reperfusion injury (IRI)^1^ occurs in approximately 30% of patients with acute myocardial infarction (AMI).^2^ The cardiac microvascular IRI is primarily caused by cardiac microcirculation endothelial cell (CMEC) apoptosis which induces endothelial swelling, microvascular spasms, and capillary obstruction and slows or stops microcirculation blood flow^3^ following the restoration of the epicardial blood flow, leading to additional myocardial damage and increased 30‐day mortality rates.^4^ These evidences suggest vasculature IRI reduces the effectiveness of reperfusion therapy and compromising the clinical benefits of patients with AMI,^5^ and therefore, strategies to protect cardiac microvascular against IRI and maintain their normal function may represent crucial adjuvant modalities for patients with AMI.

Evidence has accumulated for the protective actions of physiological concentrations of melatonin on microcirculation via maintaining the endothelial barrier function,^6^ preserving endothelial permeability,^7^ reducing cellular excessive oxidative stress,^8^ and alleviating endothelial‐dependent NO overproduction.^9^ However, the role of melatonin in cardiac microvascular damage is far from clear. Recently, mitochondrial homeostasis has emerged as the key regulator of cell death regarding to cardiac IRI, especially mitochondrial fission and mitophagy.^10^, ^11^ Excessive mitochondrial fission aggravates cardiac IRI via alternating the balance of pro‐ and antisurvival factors. In addition to mitochondrial fission, cells are capable to selectively remove several dysfunctional mitochondria from the integrated network to maintain organelle quality and homeostasis via an autophagy‐related process, termed mitophagy.^12^ An excessive shift toward mitophagy consumes most of mitochondria, leading to energy shortage and subsequent cellular death in cardiomyocytes. What remains unclear is whether mitochondrial fission and mitophagy are the trigger of cardiac microvascular IRI and CMEC death and, if so, whether melatonin is capable of alleviating cardiac microcirculatory damage by regulating mitochondrial fission and mitophagy.

AMP‐activated protein kinase signaling (AMPK) acts as an intracellular energy sensor. Previous study has found that melatonin was the trigger of AMPK pathways.^13^ Furthermore, activated AMPK by melatonin protects mitochondrial respiration.^14^ Recent studies suggested that activation of AMPK could repress mitochondrial fission via inactivation of dynamin‐related protein 1 (Drp1)^15^ and other studies indicated that AMPK could also handle mitophagy.^16^ However, whether melatonin protects cardiac microvascular against IRI through modulation of excessive mitochondrial fission and mitophagy by activation of AMPK remains unknown. To solve this problem, we generated AMPKα^−/−^ mice (AMPKα is the major isoform in endothelial cells^17^) to observe the protective effects of melatonin on mitochondria. The results suggested that melatonin protected cardiac vasculature against IRI in an AMPKα‐dependent manner. Activation of AMPKα by melatonin blunted mitochondrial fission, which prevented voltage‐dependent anion channel 1 (VDAC1) and hexokinase 2 (HK2) disassociation, mitochondrial permeability transition pore (mPTP) opening and excessive mitophagy, contributing to CMEC survival in response to IRI. Furthermore, more CMEC survival via melatonin treatment by activation of AMPKα improved the endothelial barrier function, reduced inflammatory cell infiltration, preserved e‐NOS contents, restored blood flow, limited infarcted area, and ultimately reduced cardiac microvasculature IRI.

## 2 Material and Methods

### 2.1 Animal models of cardiac IRI

This study was conducted in accordance with the Declaration of Helsinki and the guidelines of the Ethics Committee of Chinese PLA (People's Liberty Army) General Hospital, Beijing, China. All experimental protocols were approved by Ethics Committee of Chinese PLA (People's Liberty Army) General Hospital, Beijing, China. AMPKα^−/−^ mice with a C57BL/6 background were generated as previously described.^15^ AMPKα^−/−^ and wild‐type (WT) male mice (8‐wk‐old, 20‐25 g) were used to induce IRI model. For induction of IRI, a 7‐0 silk suture was subsequently passed underneath the left anterior descending coronary artery, and a slipknot was tied with 30 minutes of ischemia followed by 2‐h reperfusion. The melatonin (Sigma‐Aldrich, St. Louis, MO, USA) was administered (20 mg/kg) intraperitoneally 12 hours before IRI according to our previous study.^18^ Melatonin was initially dissolved in ethanol and then diluted in sterile water (final concentration of ethanol <1%). At the end of the reperfusion period, the hearts were stained with 2% Evans blue and 1% TTC. The infarct size was expressed as percentage of the total left ventricular (LV) area (n=6/group).

### 2.2 CMEC culture and IRI induction in vitro

CMECs were isolated from the hearts of AMPKα^−/−^ and WT male mice using the enzyme dissociation method as described by our previous study.^19^ The purity of the cultured cells was assessed by CD31 staining and uptake of acetylated low‐density lipoprotein (Fig. S1A‐B). In vitro, CMECs obtained from WT and AMPKα^−/−^ mice, respectively. As shown in Fig. S2, melatonin could activate AMPKα at Thr172 in WT‐CMECs but not in AMPKα^−/−^ ‐CMECs. Meanwhile, AMPKα gain‐of‐function experiment was performed by AMPKα adenovirus vector (Ad) overexpression in CMECs isolated from AMPKα^−/−^ mice (Ad+AMPKα^−/−^). For melatonin pretreatment, 5 μmol L^−1^ melatonin^18^ was used 12 hours before IRI in WT‐CMECs, AMPKα^−/−^‐CMECs and Ad+AMPKα^−/−^‐CMECs, which were named Mel+WT group, Mel+AMPKα^−/−^ group, and Ad+Mel+AMPKα^−/−^ group, respectively. The same volume of PBS was used in WT‐CMECs 12 hours before IRI, which was named WT group. The IRI in vitro was mimicked by 30 minutes of hypoxia with serum starvation and 2 hours of reoxygeneration.^19^ Hypoxic conditions used fresh Hanks solution with 95% N_2_ and 5% CO_2_. The pH was adjusted to 6.8 with lactate to mimic ischemic conditions.

### 2.3 Echocardiogram and myocardial contrast echocardiography (MCE)

Echocardiography was performed after 2‐h reperfusion by echocardiogram (14.0 MHz, Sequoia C512; Acuson, Germany). Myocardial contrast echocardiography (MCE) was performed using a 14‐MHz linear transducer (Acuson Sequoia C512 system) with constant infusion of microbubbles (10% Perflutren lipid microspheres (Definity, Lantheus Imaging) diluted 10‐fold in sterile saline) at 20 mL/min as previously described^20^ and with a mechanical index of 0.24. Parasternal long‐axis views were obtained in real time following the destruction of microbubbles using a sequence of 10 high‐energy frames (mechanical index 1.9). The signal intensity was determined for 10 seconds after the high‐energy sequence at a frame rate of 30 Hz. A quantitative analysis of perfusion was performed using Research‐Arena software (Tomtec, Unterschleissheim, Germany).

### 2.4 Immunohistochemistry, immunofluorescence staining, cross‐linking of VDAC1, and microvascular imaging by gelatin‐ink perfusion

Infarcted tissue immunohistochemical staining was performed on 4 mm sections using eNOS 1:200 (Abcam plc, Cambridge, MA, USA) and plasma albumin 1:500 (Abcam plc). The primary antibodies for cells or tissue immunofluorescence staining were as follows: CD31 (1:1500, Abcam plc), VE‐cadherin (1:1000, Abcam plc), Cyt‐c (1:500, Cell Signaling Technology, Inc., Danvers, MA, USA), ICAM1 (1:1000, Abcam plc), Drp1 (1:500, Cell Signaling Technology, Inc.), Hexokinase2 (1:500, Bioss, Beijing, China), and Parkin (1:1000, Cell Signaling Technology, Inc.). DAPI (Sigma‐Aldrich), lysosome stain, and a mitochondrion‐selective MitoFluor^™^ stain (Molecular Probes, Burlington, ONT, CA) were used to marker the nuclear, lysosome, and mitochondria, respectively. For the cross‐linking of VDAC1, cells were harvested and treated with DMSO as a vehicle control (2%, as used in compound‐containing samples) or cross‐linked with 0.5 mm ethylene glycolbis (succinimidyl succinate) (EGS) for 10 minutes at 30°C. The reaction was quenched by the addition of 20 mm Tris‐HCl (pH 7.4). Samples were analyzed by SDS‐PAGE followed by immunoblotting.

Gelatin‐ink perfusion was conducted following IRI. The 37°C ink plus 3% gelatin (gelatin‐ink staining) was perfused via the jugular vein, and the room temperature was maintained at 25‐30°C. When the limbs turned black, the great vessels of the cardiac base and the superior and inferior vena cava were ligated. The hearts were subsequently maintained at 4°C for at least 1 hour, then removed and fixed in 4% paraformaldehyde and processed for cryosectioning.

### 2.5 Isolation of mitochondrial‐enriched fraction and lysate preparation and mitochondrial morphology analysis

Cardiac microcirculation endothelial cells were washed with cold PBS and incubated on ice in lysis buffer (Beyotime, Beijing, China) for 30 minutes. The cells were subsequently scraped, and the homogenates were spun at 800×*g* for 5 minutes at 4°C. The supernatants were centrifuged at 10 000×*g* for 20 minutes at 4°C to acquire the pellets, which were spun again. The final pellets were suspended in lysis buffer containing 1% Triton X‐100 and were noted as mitochondrial‐rich lysate fractions.

To assess changes in mitochondrial morphology, treated cells were fixed in 4% paraformaldehyde and stained using a TOM20 primary antibody to label the mitochondria. A confocal microscope was subsequently used to capture single‐cell images, which were analyzed using ImageJ 1.47v software according to previous study.^21^ In brief, the fluorescent images were converted to binary images, and the length, area, width, and perimeter of the mitochondria were determined. The aspect ratio (AR, a measure of the length of the mitochondria) and form factor (FF, a measure of the degree of mitochondrial branching) were calculated for each cell. The minimum value for both parameters is 1, which would represent a sphere. Healthy, elongated, and interconnected mitochondria have high FF and AR values, whereas fragmented and discontinuous mitochondria have low FF and AR values.^22^

### 2.6 Mitochondrial membrane potential (ΔΨm) measurements and LDH assay

The mitochondrial transmembrane potential was analyzed using a JC‐1 Kit (Beyotime) and LDH release was measured via a LDH release kit (Beyotime) as we previously described^23^.

### 2.7 Apoptosis detection and mPTP opening

Apoptosis was detected using a TUNEL assay. The opening of the mPTP was visualized as a rapid dissipation of tetramethylrhodamine ethyl ester (TMRE) fluorescence. Arbitrary mPTP opening time was determined as the time when TMRE fluorescence intensity decreased by half between initial and residual fluorescence intensity according to previous study.^24^

### 2.8 Western blot, co‐immunoprecipitation, and electron microscopy

The primary antibodies for Western blots were as follows: VDAC1 (1:1500, Abcam plc), pDrp1‐616 (1:500, Cell Signaling Technology, Inc.), pDrp1‐637 (1:500, Abcam plc), HK2 (1:500, Bioss), Parkin (1:1000, Cell Signaling Technology, Inc.), PINK1 (1:1000, Cell Signaling Technology, Inc.), LC3I (1:1000, Cell Signaling Technology, Inc.), and LC3II (1:1000, Cell Signaling Technology, Inc.).

For co‐immunoprecipitation experiments, proteins from CMEC were cross‐linked in 1% paraformaldehyde followed by washing in PBS containing 100 mmol L^−1^ glycine. The immunoprecipitates were loaded on SDS‐PAGE and probed with HK2 antibody.

For electron microscopy, samples were dehydrated using acetonitrile and graded methanol, embedded in epoxy resin (EMbed‐812; Electron Microscopy Sciences, USA) and polymerized at 70°C overnight. Hitachi H600 Electron Microscope (Hitachi, Japan) was used to capture the images.

### 2.9 Construction of adenovirus (AD) for AMPKα overexpression

For overexpression of AMPKα, the pDC316‐mCMV‐AMPKα plasmid was purchased from Vigene Bioscience and was transfected with framework plasmid (1:1) into 293 T cells using Lipofectamine 2000. After transfection for 48 hours, the viral supernatant was collected and identified by PCR. Following amplification, the supernatant was acquired again and filtered through a 0.45‐μm filter to obtain the Ad‐AMPKα. The transfection efficiency results are presented in Fig. S3A‐B.

### 2.10 Statistical analysis

The data are described as the mean±standard deviation (SD) of at least three independent experiments and were analyzed by one‐way analysis of variance (ANOVA). The limit of statistical significance between the treated and control groups was *P*<.05.

## 3 Results

**Figure 1:**
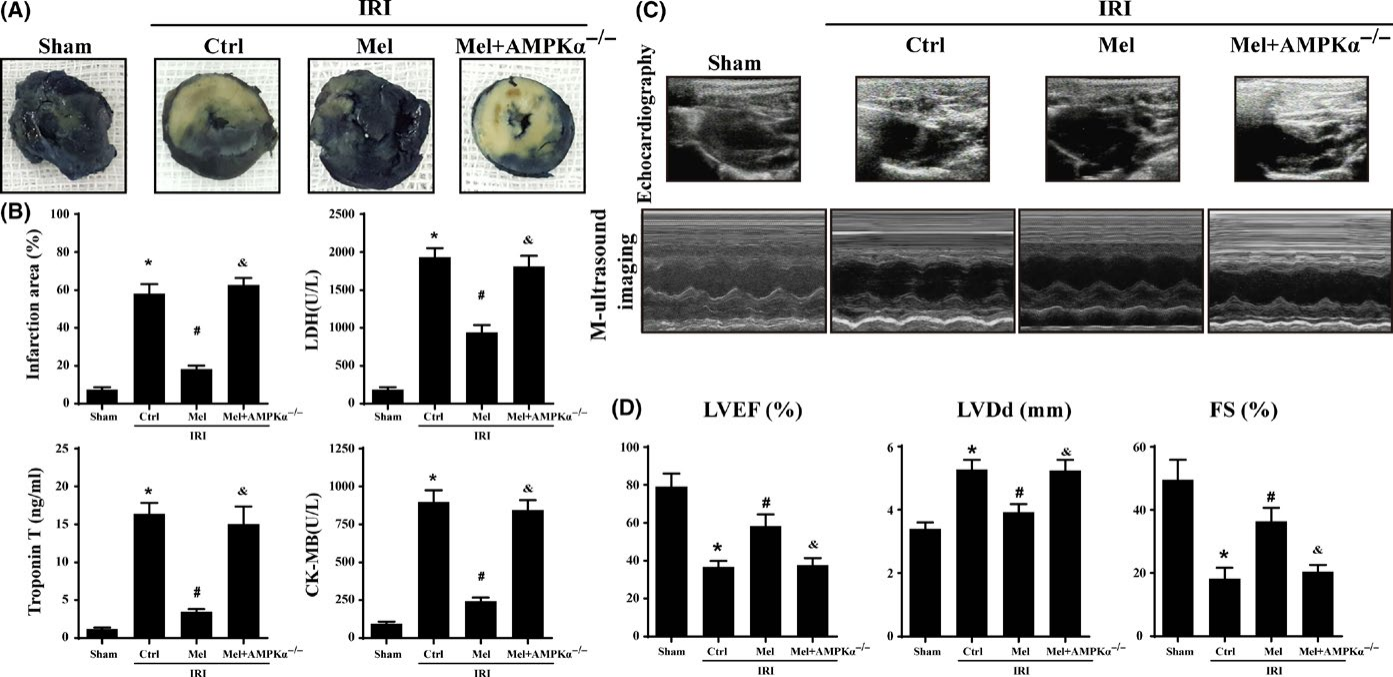
Melatonin reduced infarct size and preserved cardiac function following ischemia/reperfusion injury in vivo which was performed by 30 min of ischemia followed by 2‐h reperfusion (n=6/group). (A). Representative pictures of heart sections with TTC and Evans Blue staining. (B). Bar graph indicates the infarct size expressed as a percentage of the total left ventricular area. The lactate dehydrogenase release, troponin T contents, and CK‐MB values were assessed via ELISA. (C). Representative M‐mode echocardiograms was performed after 2‐h reperfusion with the parasternal long‐axis views in each group. (D). Quantitative analysis of cardiac function by echocardiography. *P<.05 vs sham group; #*P*<.05 vs Ctrl‐IRI group, &*P*<.05 vs Mel‐IRI group

As shown in Figure 1A, mice treated with melatonin developed smaller infarcts expressed as a percentage of the total left ventricular (LV). However, loss of AMPKα, the beneficial effects of melatonin on infarct size disappeared. Furthermore, lactate dehydrogenase (LDH), troponin T, and creatine kinase‐MB (CK‐MB) expression were obviously increased in response to IRI and melatonin reduced LDH release, troponin T contents, and CK‐MB in WT mice but not in AMPKα^−/−^ mice (Figure 1B). Furthermore, melatonin also improved cardiac function in the context of IRI as evidenced by an improvement in left ventricular ejection fraction (LVEF), left ventricular diastolic dimension (LVDd), and left ventricular fractional shortening (LVFS). However, AMPKα depletion reduced the protective actions of melatonin on cardiac function. (Figure 1C‐D).

**Figure 2:**
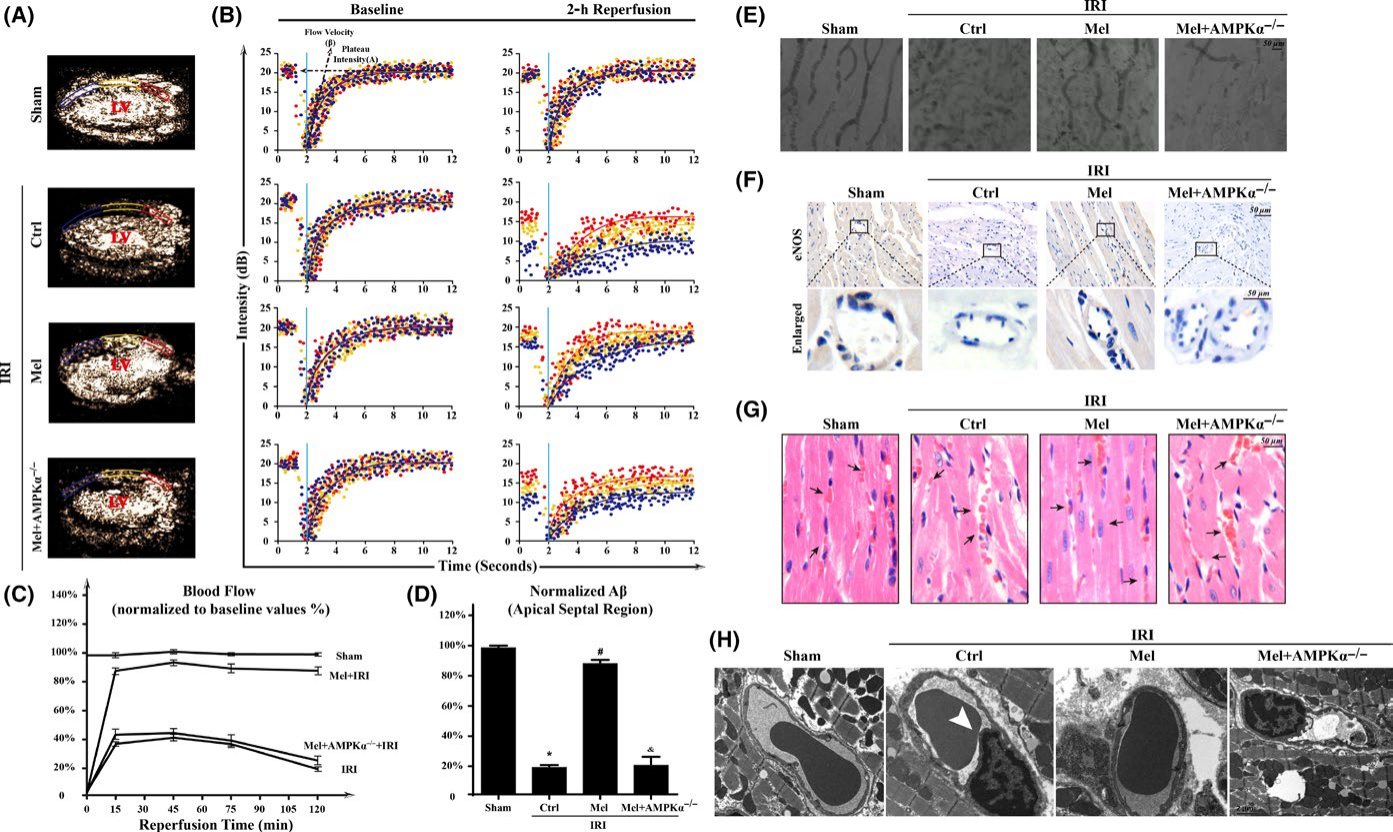
Melatonin protected acute microcirculation malfunction during ischemia/reperfusion injury(IRI) via AMPKα. (A‐B). Representative case of a myocardial contrast echocardiography myocardial contrast echocardiography parasternal long‐axis image, which was divided into three regions: apical septum (blue), mid‐septum (yellow), and basal septum (red). Left ventricular: Left ventricle. A: plateau intensity, β: flow velocity. (C). Myocardial blood flow (A×β) profiles in the apical region for different groups at 15, 45, 75, and 120 min after reperfusion. (D). Apical myocardial blood flow 120 min after reperfusion. n=6 per group. (E). Microvascular image detection by ink staining. (F). Immunohistochemistry of endothelial nitric oxide synthaseS expression. (G). HE staining for red blood cell morphology in different groups. (H). Transmission electron microscopy (TEM) was used to observe the structural changes of microvessel in response to IRI, including microvascular wall destruction and luminal stenosis (white arrow) of CMEC. *P<.05 vs sham group, #*P*<.05 vs Ctrl‐IRI group, &*P*<.05 vs Mel‐IRI group

Myocardial contrast echocardiography was used to assess the changes in microvascular perfusion following IRI. The melatonin treatment caused smaller perfusion defect zones at the 2‐h time point of reperfusion in WT mice but not in AMPKα^−/−^ mice (Figure 2A, B,C and D) are representative region‐specific replenishment curves showing the myocardial blood flow parameters A (plateau intensity) and β (flow velocity) and the myocardial blood flow (MBF) profiles (A×β) at 2 hours postreperfusion. These data suggest that the myocardial blood flow is increased in response to melatonin administration in a AMPKα‐dependent manner. Furthermore, microvascular imaging using gelatin ink (Figure 2E) also indicated that IRI interrupted the bloodstream, whereas the melatonin application recovered the patency of the microvascular blood flow in WT mice but not in AMPKα^−/−^ mice. Besides, IRI substantially decreased the expression of endothelial nitric oxide synthase (eNOS), whereas the treatment with melatonin increased the eNOS levels via AMPKα (Figure 2F). Additionally, IRI also induced the accumulation of irregularly formed red blood cells (RBC) as a result of turbulent flow during IRI conditions (Figure 2G). However, melatonin treatment reversed RBCs morphology exhibiting a regular shape that was similar to a “parachute” or “arrow” in WT mice rather than AMPKα^−/−^ mice. Moreover, results from transmission electron microscope (TEM) also displayed that IRI evoked the luminal stenosis. The surface of the cardiac microvessels was smooth and well‐integrated in melatonin‐treated WT mice but not AMPKα^−/−^ mice (Figure 2H).

**Figure 3:**
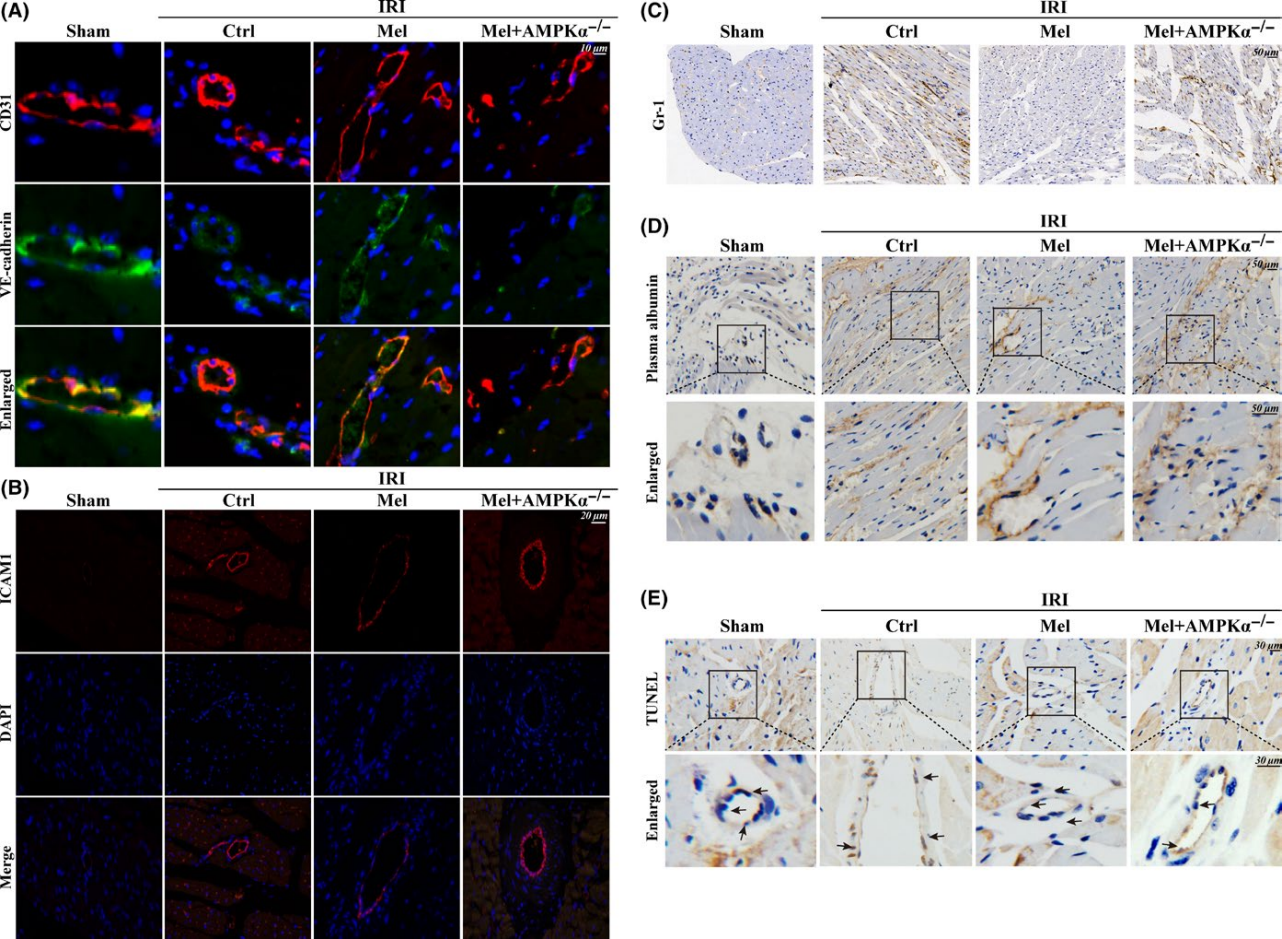
Melatonin maintained microvessels endothelial barrier integrity, reduced vascular permeability, and alleviated cellular death. (A). The endothelial barrier integrity was assessed via VE‐cadherin staining. Discontinuous punctiform or linear expression of VE‐cadherin could be observed in the ischemia/reperfusion injury(IRI) group indicative of the broken endothelial barrier. However, melatonin could reverse the continuous linear of VE‐cadherin fluorescence via AMPKα. (B). The expression of ICAM1 in cardiac microcirculation endothelial cells(CMEC). (C). The Gr‐1^+^ neutrophil infiltration into the myocardial tissues. (D). The leakage of plasma albumin out of the surface of the vessel wall into interstitial spaces suggested the increased microvascular permeability in response to IRI. (E). TUNEL assay to assess CMEC death

Ischemia/reperfusion injury induced the downregulation of VE‐cadherin, a junctional protein that sustains microvascular barrier. While melatonin forcefully maintained continuous VE‐cadherin fluorescence in WT mice but not in AMPKα^−/−^ mice. (Figure 3A). Furthermore, melatonin could also attenuate IRI‐enhanced ICAM‐1 expression on the surface of microvascular in WT mice but not in AMPKα^−/−^ mice (Figure 3B). The collapse of endothelial barrier was followed by more Gr‐1^+^ cells in the myocardial tissues (Figure 3C). However, melatonin deeply reduced Gr‐1^+^ neutrophil infiltration into the intima. Moreover, IRI also induced more plasma albumin leakage from the vessel. While melatonin weakened the diffusion of the plasma into the outer surface of the vessel wall (Figure 3D) in WT mice but not AMPKα^−/−^ mice. Furthermore, the destruction of microvessel integrity and barrier function may be also the results of CMEC death and TUNEL assay (Figure 3E) confirmed that melatonin could protect CMEC against IRI‐mediated death via activation of AMPKα^−/−^.

**Figure 4:**
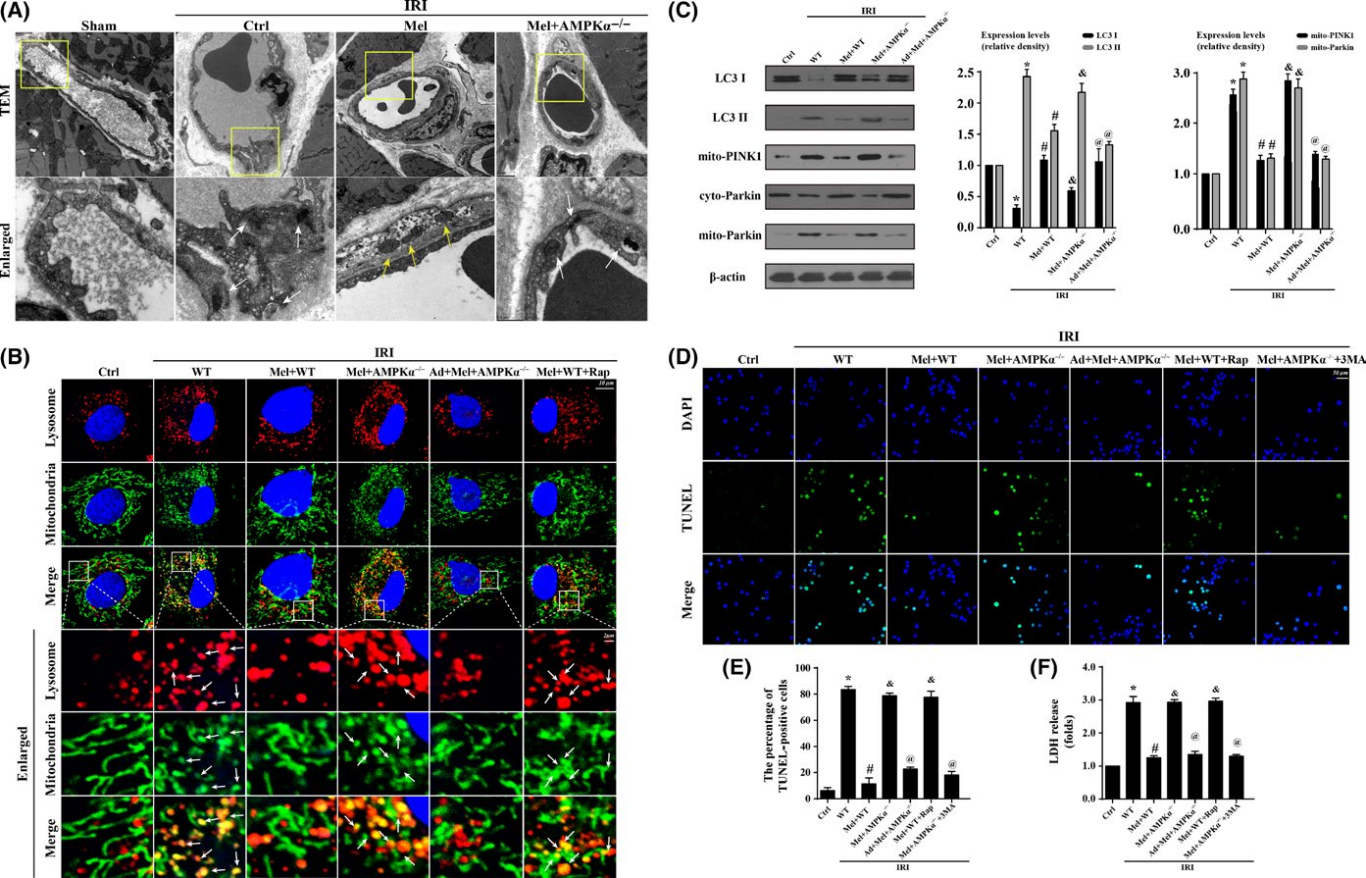
Ischemia/reperfusion injury induced(IRI) cardiac microcirculation endothelial cells(CMEC) death via excessive mitophagy in vitro. Wild‐type mice‐ and AMPKα^−/−^ mice‐derived CMEC treated with melatonin were named Mel+WT and Mel+AMPKα^−/−^ group, respectively. Furthermore, AMPKα gain‐of‐function experiments was performed in CMECs from AMPKα^−/−^ using adenovirus vector following melatonin treatment, named Ad+Mel+AMPKα^−/−^ group. Meanwhile, rapamycin (Rap), an activator of mitophagy, was used in Mel+WT as the positive control group. The IRI in vitro was mimicked by 30 min of hypoxia with serum starvation and 2 h of reoxygeneration. (A). Transmission electron microscope was performed to observe mitophagy in vivo. IRI induced more mitochondria were contained by lysosome, and these changes were reversed by melatonin. Yellow arrow suggested the normal mitochondria. White arrow indicated the digested mitochondria or fragmented mitochondria in lysosome. (B). The co‐location of lysosome and mitochondria. More fragmented mitochondria were consumed by lysosomes in response to IRI, which was reversed by melatonin. Rap reduced more numbers of mitochondria contained in lysosome. (C). The change of proteins related to mitophagy. IRI increased the ratio of LC3 II/LC3 I via PINK1/Parkin pathway activation. (D‐E). TUNEL staining demonstrated that mitophagy evoked CMEC death. (F). The lactate dehydrogenase release assay to detect cellular damage. 3‐MA, an inhibitor of mitophagy. *P<.05 vs Ctrl group, #*P*<.05 vs IRI‐WT group, &*P*<.05 vs IRI‐Mel+WT group, @*P*<.05 vs IRI‐Mel+ AMPKα^−/−^ group

To explore the mechanism by which melatonin protected cardiac microvascular against IRI, we observed mitophagy via TEM in vivo. In response to IRI, mitochondria in microvasculature became smaller and punctate. Furthermore, some mitochondria or fragmented mitochondria was contained in lysosome suggestive of mitophagy in context of IRI (white arrow in Figure 4A). In contrast, melatonin treatment protected mitochondria against lysosome digestion in WT mice but not in AMPKα^−/−^ mice (yellow arrow in Figure 4A). Furthermore, in vitro, we found the overwhelming majority of fragmented mitochondria were consumed by lysosomes after IRI (Figure 4B), which was evidenced by the increased co‐localization of mitochondria and lysosomes. However, these changes were reversed by melatonin. Similarly, treatment with rapamycin (Rap), an activator of mitophagy, also increased the number of mitochondria consumed by lysosomes. However, loss of AMPKα suppressed the inhibitory effects of melatonin on mitophagy.

**Figure 5:**
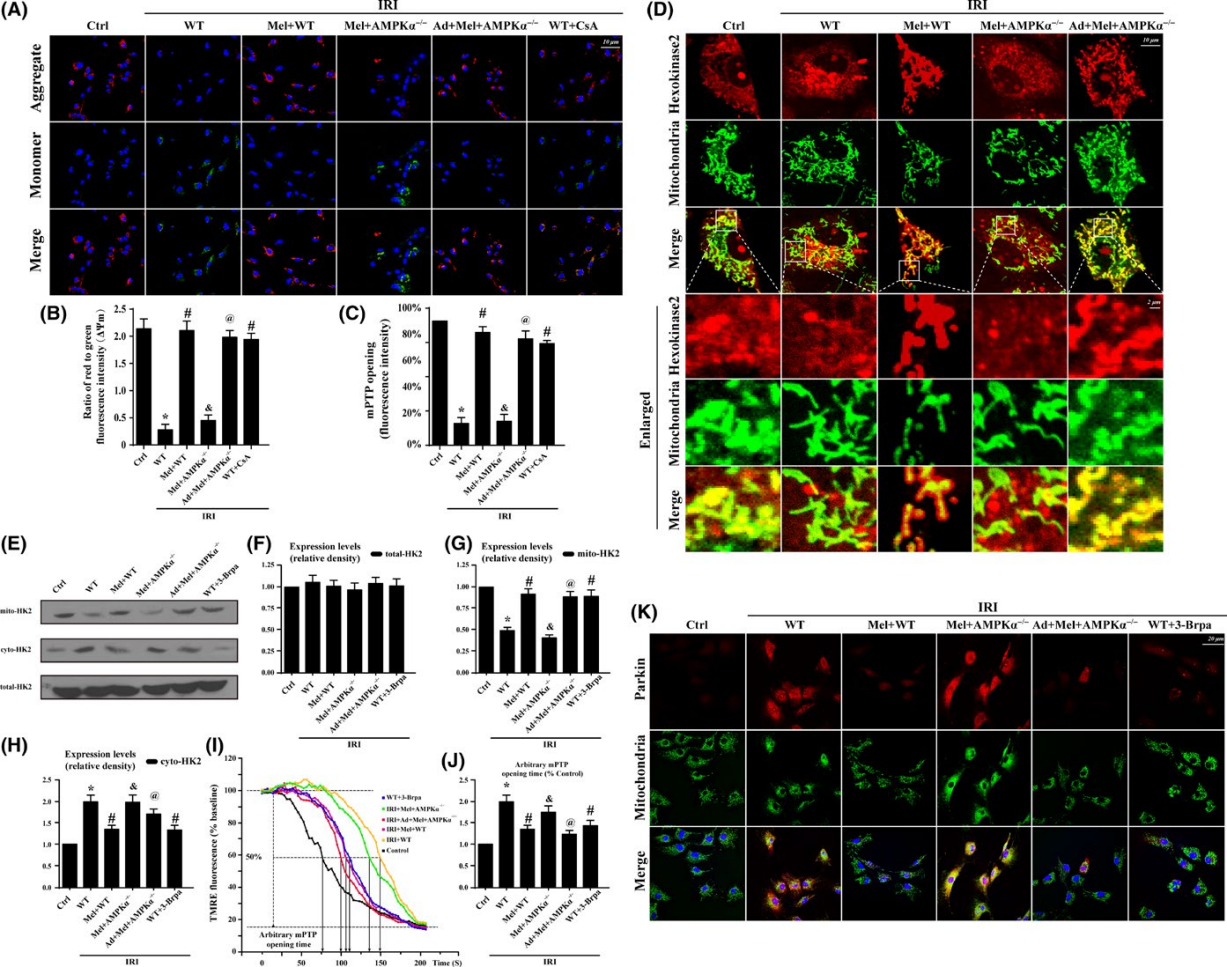
Mitophagy was activated by mitochondrial permeability transition pore(mPTP) opening that was induced by hexokinase 2(HK2) separation from the mitochondria in response to Ischemia/reperfusion injury induced(IRI). (A‐B). The change of membrane potential (∆Ψm) by JC‐1 staining. (C.) Change in mPTP opening. (D‐H). The subcellular location of HK2 via immunofluorescence and Western blots. IRI contributed to the HK2 separation from the mitochondria to the cytoplasm, which was reversed by melatonin via AMPKα^−/−^. However, IRI or melatonin had no effects on the total content of HK2 but influenced its subcellular distribution between the mitochondria and cytoplasm. (I‐J). Arbitrary mPTP opening time by tetramethylrhodamine ethyl ester(TMRE) fluorescence of 3–Brpa which is the inhibitors of V HK2 interaction. Arbitrary mPTP opening time was determined as the time when the TMRE fluorescence intensity decreased by half between the initial and residual fluorescence intensity. CsA, an mPTP blocker, was used as the negative control. (K). The co‐expression of Parkin and mitochondria. Inhibition of HK2 liberation reduced the interaction of Parkin with mitochondria. *P<.05 vs Ctrl group; #*P*<.05 vs IRI‐WT group, &*P*<.05 vs IRI‐Mel+WT group, @*P*<.05 vs IRI‐Mel+ AMPKα^‐/‐^ group

To explain the mechanism by which IRI induced mitophagy, we focused on the PINK1/Parkin pathway. Figure 4C displayed that IRI increased the content of mitochondrial PINK1 and subsequent Parkin translocation from the cytoplasm to mitochondria, which was parallel to the shift from LC3I to LC3II. However, melatonin suppressed PINK1 and Parkin accumulation on mitochondrial and further terminated the LC3I to LC3II shift in an AMPKα‐involved manner. At last, we found IRI‐mediated cellular apoptosis could be reversed by mitophagy inhibition or melatonin treatment in an AMPKα‐dependent manner via TUNEL assay (Figure 4D‐E). Besides, LDH assay also supported such results (Figure 4F). The mPTP opening and ΔΨm dissipation play a decisive role in activating PINK1/Parkin/mitophagy pathways. In this study, IRI led to the complete dissipation of ΔΨm and mPTP opening (Figure 5A‐C), which was reversed by melatonin via AMPKα. Moreover, cyclosporin a (CsA), an inhibitor of mPTP opening, was used as the negative control group (Figure 5A‐C).

Furthermore, the opening of mPTP can be inhibited by HK2^25^ binding to mitochondria via structurally interfering with the ability of pro‐apoptotic proteins such as Bax and Bad to translocate from the cytoplasm to mitochondria.^26^ Therefore, we observed the changes in HK2 under melatonin treatment. The results in Figure 5D suggested that IRI caused lower levels of mitochondrially bound HK2 but higher levels of cytoplasmic HK2. In the enlarged image (Figure 5D), fragmented or swollen mitochondria captured less HK2 in response to IRI, while melatonin promoted HK2 remigration to the mitochondria via AMPKα. Interestingly, despite the decrease in mitochondrial‐related HK2, there was no change in the total content of HK2 in CMECs after IRI (Figure 5E‐H), suggesting that IRI only influenced the subcellular distribution of HK2. To establish the role of HK2 liberation in mPTP‐dependent mitophagy, 3‐bromopyruvate (3‐Brpa), an inhibitor of HK2 liberation, was used. We found that the modification of HK2 binding to mitochondria by melatonin via AMPKα not only improved ΔΨm (Figure 5I‐J) but also reduced Parkin translocation on mitochondria (Figure 5K).

**Figure 6:**
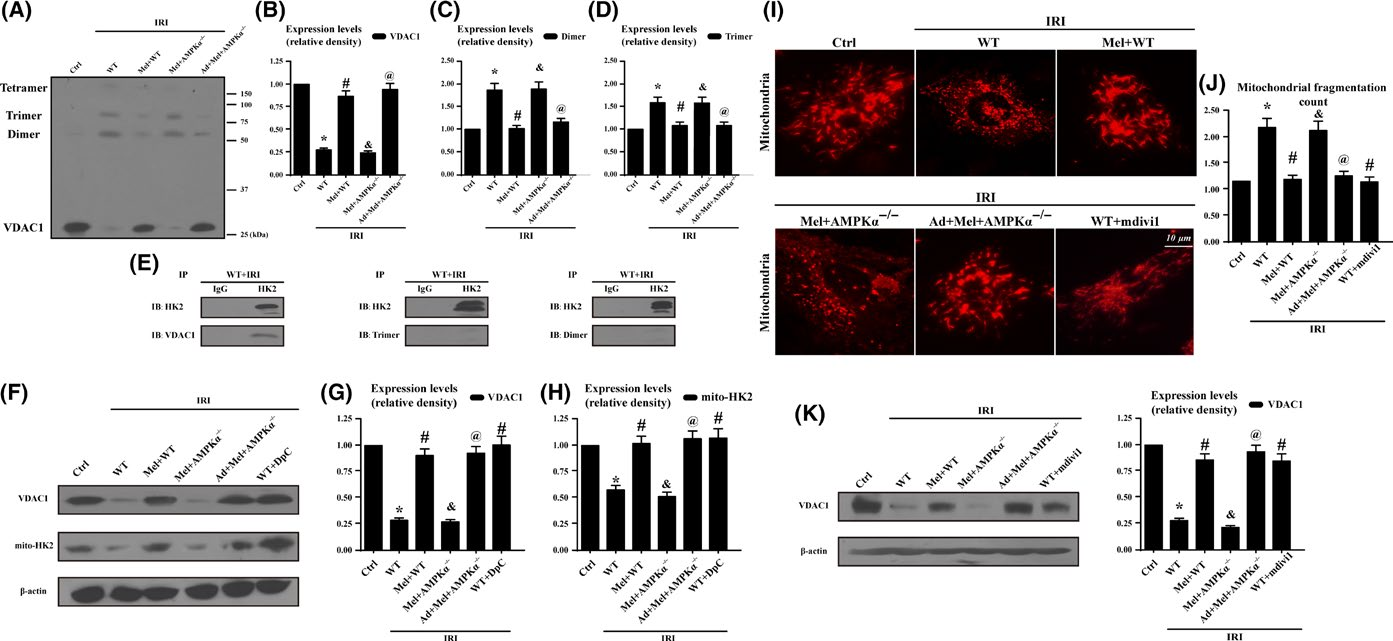
Hexokinase 2(Hk2) liberation was attributed to mitochondrial fission‐mediated voltage‐dependent anion channel 1(VDAC1) oligomerization which was inhibited by melatonin via AMPKα. (A‐D). The evaluation of VDAC1 oligomerization via EGS‐based cross‐linking and immunoblotting using anti‐VDAC1 antibodies. Ischemia/reperfusion injury induced increased VDAC1 oligomerization corresponding to molecular masses of 69 and 95 kDa, whereas melatonin can reverse this change. (E). HK2 and VDAC1 interaction assessed by immunoprecipitation (IP) experiments. (F‐H). Inhibition of VDAC1 oligomerization could increase the contents of mitochondria‐HK2. Diphenylamine‐2‐carboxylic acid (DpC), an inhibitor of VDAC1 oligomerization, was used as the negative control group. (I‐J). Mitochondria of cardiac microcirculation endothelial cells were labeled with anti‐Tom20 antibody to determine the number of cells with mitochondria fragmentation. To assess changes in mitochondrial morphology quantitatively, the aspect ratio (AR, the mitochondrial length) and form factor (FF, the degree of mitochondrial branching) were calculated for each cell (the minimum value for both parameters is 1). High FF and AR values shown healthy mitochondria whereas low FF and AR indicated fragmented mitochondria. (K). Inhibition of mitochondrial fission could reverse the VDAC1 expression. Mdivi1, an inhibitor of mitochondrial fission, was used as the negative control group. *P<.05 vs Ctrl group; #*P*<.05 vs IRI‐WT group, &*P*<.05 vs IRI‐Mel+WT group, @*P*<.05 vs IRI‐Mel+ AMPKα^−/−^ group. VDAC1, voltage‐dependent anion channel1; HK2, hexokinase2

Mitochondrial VDAC1 tightly couple the cytoplasmic HK2 with the mitochondria to prevent mPTP opening via the suppression of the Bax/Bad interaction with mitochondria.^27^ However, IRI significantly reduced VDAC1 expression but increased the percentage of VDAC1 oligomerization products with molecular masses of 69 and 95 kDa (Figure 6A‐D), which was reversed by melatonin via AMPKα. To explore whether the oligomerization state of VDAC1 influenced the liberation of HK2 into the cytoplasm, we focused on protein interactions between HK2 and VDAC1 or VDAC1 dimers and multimers. Our results showed an interaction between VDAC1 and HK2 but not between oligomeric VDAC1 and HK2 (Figure 6E). To further provide evidence for the role of VDAC1 oligomerization and subsequent HK2 liberation, we used diphenylamine‐2‐carboxylic acid (DpC) to inhibit VDAC1 oligomerization. It seems likely that inhibiting VDAC1 oligomerization may prove as an effective intervention for enhancing HK2 anchored to mitochondria (Figure 6F‐H), which was similar to the results in the melatonin groups. To explore the mechanism by which melatonin reduced VDAC1 oligomerization, we focused on mitochondrial fission. Firstly, the mitochondria in melatonin group had fewer free ends than those in the IRI group (Figure 6I‐J). Secondly, inhibition of fission by Mdivi1 could recover the contents of VDAC1 downregulated by IRI, which was comparable to the results in melatonin treatment. (Figure 6K).

**Figure 7:**
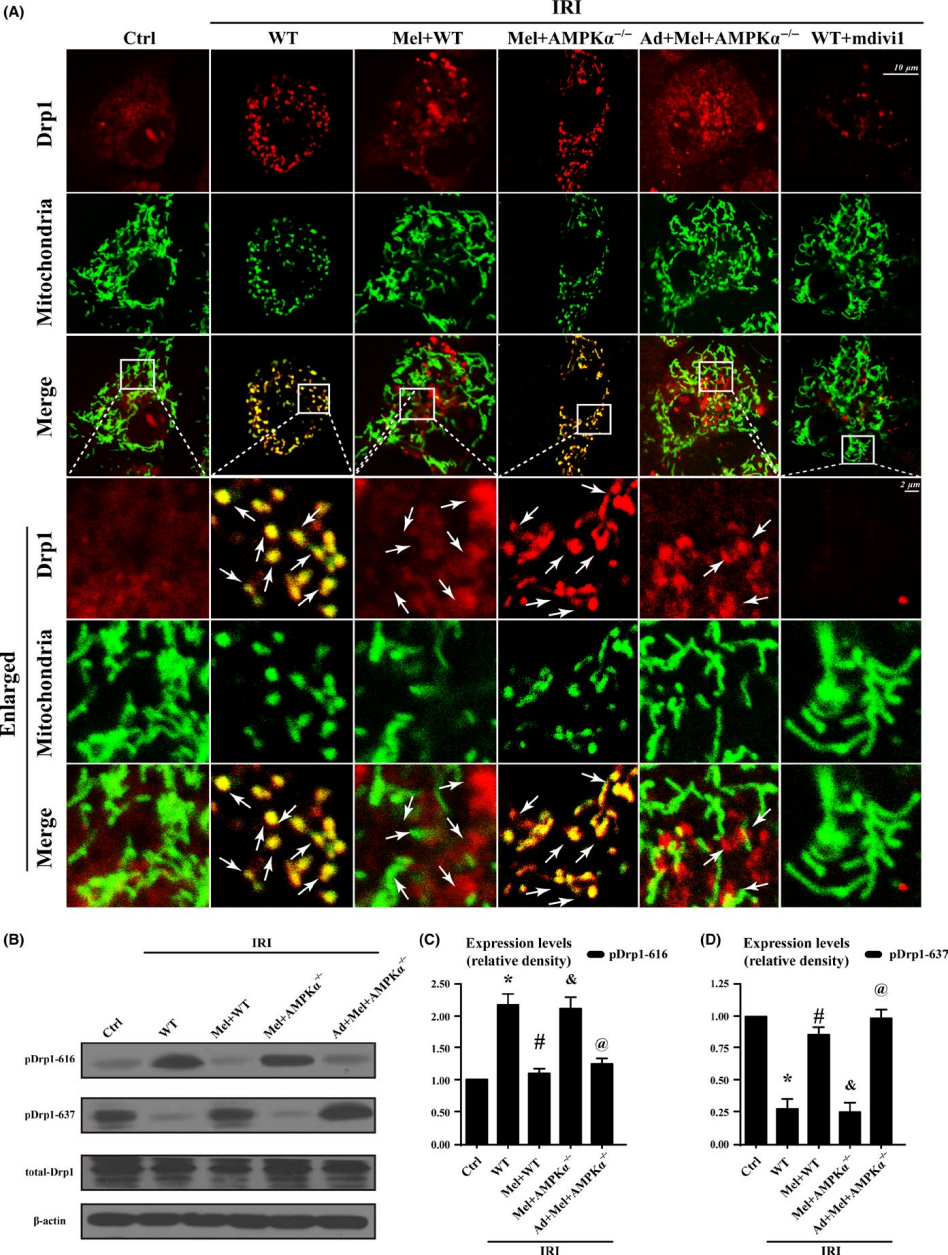
Ampkα was activated by melatonin contributed to Drp1 modifications and subsequent mitochondrial fission. (A). Co‐localization of Drp1 and mitochondria. Boxed area under each micrograph represents the amplification of the white square. More Drp1 was located on fragmented mitochondria while melatonin could reduce Drp1 migration on mitochondria via AMPKα. White arrow indicated the aggregated Drp1 on mitochondria or not. (B‐D). Activated AMPKα was involved in regulating Drp1 phosphorylation. *P<.05 vs Ctrl group; #*P*<.05 vs IRI‐WT group, &*P*<.05 vs IRI‐Mel+WT group, @*P*<.05 vs IRI‐Mel+ AMPKα^−/−^ group

Drp1 shuttling between the cytoplasm and the mitochondrial surface is indispensable to mitochondrial fission. Figure 7A exhibited that IRI significantly increased the overlap of Drp1 and mitochondria, and the results also demonstrated that the mitochondria marked by Drp1 had greater amounts of free debris. However, melatonin reduced Drp1 foci on the mitochondria with almost normal morphology and fewer fragments (white arrow in Figure 7A). Loss of AMPKα reduced the protective actions of melatonin on mitochondrial fission. Furthermore, IRI increased Drp1 Ser616 phosphorylation but also reduced Ser637 phosphorylation (Figure 7B‐D), which was accompanied by greater amounts of Drp1 accumulation on mitochondria. In contrast, melatonin enhanced Ser637 but attenuated Ser616 phosphorylation of Drp1, followed by the lower mitochondrial Drp1 content. Moreover, loss of AMPKα abolished the effects of melatonin on Drp1 phosphorylation modification.

## 4 Discussion

**Figure 8:**
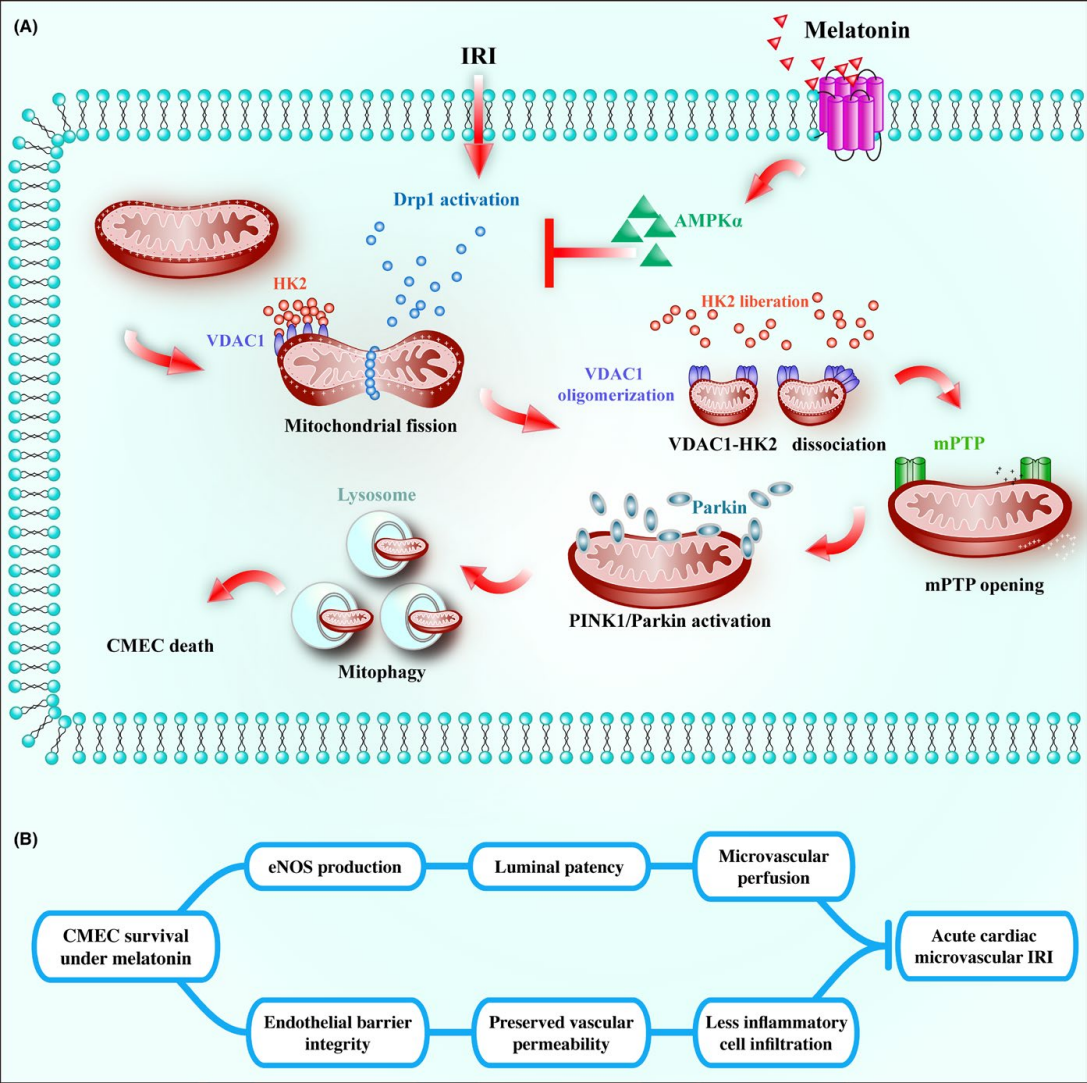
(A) Ischemia/reperfusion injury induced(IRI) activated Drp1‐dependent mitochondrial fission, which subsequently induced voltage‐dependent anion channel(VDAC1) oligomerization, hexokinase 2 liberation, mitochondrial permeability transition pore(mPTP) opening, PINK1/Parkin upregulation, and ultimate mitophagy‐mediated cardiac microcirculation endothelial cells(CMEC) death. However, melatonin strengthened CMEC survival via activation of AMPKα, followed by p‐Drp1^S637^ upregulation and p‐Drp1^S16^ downregulation, which blunted Drp1‐dependent mitochondrial fission. Suppression of mitochondrial fission by melatonin recovered VDAC1‐HK2 interaction that prevented mPTP opening and PINK1/Parkin activation, eventually blocking mitophagy‐mediated cellular death. (B) Finally, the survival of CMEC preserved barrier integrity, reduced vascular permeability, alleviated neutrophil infiltration and plasma albumin leakage, increased endothelial nitric oxide synthase contents, improved microvessel patency and contributed to microvascular perfusion, protecting cardiac microcirculatory against IRI

In the present study, we found that (i) melatonin contributes to scar restriction, blood flow recovery, microcirculatory perfusion patency, endothelial barrier integrity, eNOS production, and CMEC survival in response to IRI; (ii) melatonin inactivates Drp1 via AMPKα‐involved Drp1 post‐transcriptional modification, which is associated with the less mitochondrial fission; (iii) suppression of fission alleviates HK2 dissociation from the mitochondria via the inhibition of VDAC1 oligomerization, blocking mPTP opening, and preserving ΔΨm; and (iv) higher ΔΨm and less opened mPTP blunt PINK1/Parkin/mitophagy pathways, ultimately promoting CMEC survival. To the best of our knowledge, this is the first study to describe the role of melatonin in cardiac microvascular IRI, which regulates mitochondrial structure and function referring to mitochondrial fission, mitophagy, mPTP opening, and HK2‐VDAC1 interaction. (Figure 8A‐B).

During IRI, endothelial damage is detectable early in the course of the disease compared with the pathological changes that occur in cardiomyocytes.^28^ Furthermore, endothelial damage plays a decisive role in the pathogenesis of microvascular and cardiomyocytes complications in response to IRI. Insufficient eNOS production is a prominent feature of limited microvessel diastolic function. Besides, endothelial hyperpermeability and junctional loss derived from excessive CMEC death cause barrier malfunction, leading to adverse leukocyte adhesion and micro‐thrombus formation, which participate in vascular clotting and lumen loss. Finally, blood flow decreases due to capillary occlusion resulting from damage to the microvascular structure and function.^29^ In the present study, we first provide evidence supporting a direct defensive role for melatonin in cardiac microvascular IRI through reversing vascular dysfunction and alleviating CMEC death. In detailed, melatonin improved eNOS production and barrier function, which alleviated luminal stenosis and inflammatory cell or RBC attachment on microvessels. And these beneficial actions may be owing to melatonin's pro‐survival effect on CMEC. Through these mechanisms, melatonin increased blood circulation and cardiac perfusion, contributing to the recovery of myocardial function.

We determined that antimitophagy was the primary mechanisms of defense that was inhibited by melatonin to preserve the CMEC survival in a state of IRI. Many studies have found that during IRI, the destruction of mitochondria caused the impairment of aerobic respiration in cardiomyocytes, leading to energy exhaustion and cellular death. In the present study, we found that overwhelming majority of mitochondria was swallowed by lysosome to proceed mitophagy, which was accompanied with numerous dead cells. In fact, excessive mitophagy consumes most of mitochondria, leading to the rapid decrease in organelle abundance. The dyshomeostasis of mitophagy is coupled with the cellular energy shortage, lessening the cellular resistance to IRI. These may be the nature molecular processes underlying mitophagy‐involved cellular death. Based upon our present study, the major stimuli that drove mitophagy were the upregulated PINK/Parkin in response to IRI. Furthermore, the increased PINK/Parkin activity was attributed to lower membrane potential and more mPTP opening.^30^ Inhibition of mPTP opening not only stabilized ΔΨm but also impaired PINK1/Parkin settling on mitochondria. These data suggest that cellular death is initiated by PINK/Parkin/mitophagy that resulted from mPTP opening in response to IRI, which is entirely different from the mPTP‐mediated classical mitochondrial‐death pathway. As previously reported by our research, the classical mitochondrial‐death pathway was characterized by the cytochrome c releases from mitochondria into cytoplasm where it acts with caspase9 to inactive caspase3.^23^, ^31^ However, it is necessary to point out that there is an upstream convergence of such two death pathways and that is the mPTP opening. On one hand, mPTP opening provides a channel for cytochrome c leakage. On the other hand, mPTP opening causes ΔΨm collapse that attracts PINK/Parkin to evoke mitophagy. Our study further bolsters the role of mPTP in cellular death, which offering a crucial target to regulate IRI in clinical applications.

From another perspective, we found that melatonin suppressed mPTP opening through enhancing HK2‐mitochondria association. The interaction of HK2 with mitochondria is known to protect against cell death in many cell types^32^, ^33^ and mPTP opening is inhibited by the interaction with HK2.^25^ In the present study, IRI boosted the dissociation of HK2 from mitochondria, leading to the excessive opening of mPTP. However, melatonin augmented the tight interconnection between HK2 and mitochondria. Interestingly, there was no change in the total expression of HK2 in CMEC under melatonin treatment. Therefore, it is reasonable to hypothesize that the increase in the content of mitochondrial‐HK2 is mediated by its higher affinity with mitochondria with melatonin. It has been shown that HK2 binds to the outer mitochondrial membrane^34^, ^35^ via VDAC1, which is dynamically oligomerized.^27^ We provided evidence that VDAC1 oligomers induced by IRI had a lower affinity with HK2 while melatonin hampered VDAC1 oligomerization to guarantee HK2 tight coupling on mitochondria.

Previous study has recommended that mitochondrial fission could affect mitochondria‐related proteins oligomerization including OPA.^36^, ^37^ In this study, our findings confirmed the above conclusion. IRI caused VDAC1 oligomerization via mitochondrial fission. Melatonin could alleviate mitochondrial fission via balancing Drp1 phosphorylation by AMPKα. Notably, Drp1 has two key phosphorylation sites including Ser616 and Ser637. Drp1 could be activated by phosphorylation at Ser616 but inactivated by phosphorylation at Ser637. The balance of such two phosphorylation sites determines the activity of Drp1. In the present study, melatonin reduced Ser616 phosphorylation but increased Ser637 phosphorylation via AMPKα. These findings are consistent with previous study,^15^ suggesting the importance of direct post‐transcriptional modification effects of AMPKα on Drp1 and mitochondrial fission. In fact, several studies have demonstrated the important role of AMPKα in mitochondrial fission and mitophagy. Deletion of AMPKα has been reported to trigger excessive mitochondrial fission.^38^ In aorta endothelial cells, Drp1 and mitochondrial fission are inactivated via phosphorylation at Ser637 by AMPK.^15^ Furthermore, loss of AMPK also regulated mitophagy activity.^39^ In cardiac aging, AMPK could accentuate myocardial remodeling and contractile dysfunction via suppression of myocardial autophagy.^40^ Notably, several studies also found that mitochondrial dysfunction could activate the AMPK signaling to promote cell survival.^41^ These data indicate that the approach of activation of the AMPKα could serve as a future focal point to attenuate IRI and increase patients’ benefits from reperfusion therapy after AMI. Collectively, the results of our report illustrated the important role of mitochondrial fission in the IRI signals transmitted via the VDAC1‐HK2‐mPTP axis, which fatally triggers mitophagy cascade reactions to direct cellular death, leading to the destroy of cardiac microvascular structure and function in vivo during IRI. These data also offer an easy and effective method to intervene against cardiac vasculature IRI. This method is the use of melatonin. However, more insight into this combination should be obtained to provide ample evidence for clinical applications.

There are some limitations in the present study. Firstly, our experiments were particularly focused on microvascular injury. Further investigation was required to support the present findings applied to cardiomyocytes. Whether or not the present findings can be applied to cardiomyocytes IRI warrants further investigation. Second limitation of this study is all experiments were done in global AMPKα knockout mice. Whether the effect reported in isolated CMECs is a result of immediate knocking down of AMPKα and/or indirect effects from AMPKα deletion in other organs/tissues that may also affect CMECs is an important limitation to involve the role of AMPKα activation as reported in this study. The effects of endothelial cell‐specific AMPKα deletion will be very useful in defining the relative contributions of endothelial cell AMPKα to cardiac microvascular IRI.

## Disclosure

The authors have declared that they have no conflict of interests.

## Author Contributions

HZ, YZ, and SYH involved in conception and design, performance of experiments, data analysis and interpretation, and manuscript writing; CS, PJZ, and QM involved in data analysis and interpretation; QHJ, FC, FT, and YDC involved in conception and design, data analysis and interpretation, financial support, and final approval of manuscript.
