# Supplementary material for: Melatonin protects cardiac microvasculature against ischemia/reperfusion injury via suppression of mitochondrial fission‐VDAC1‐HK2‐mPTP‐mitophagy axis
Source: J Pineal Res. 2017 Apr 27;63(1):e12413. doi: 10.1111/jpi.12413 (PMC5518188; doi:10.1111/jpi.12413)
Supplement: Supplementary file 1 [file JPI-63-na-s001.docx]

**Supplemental Figures：**

**
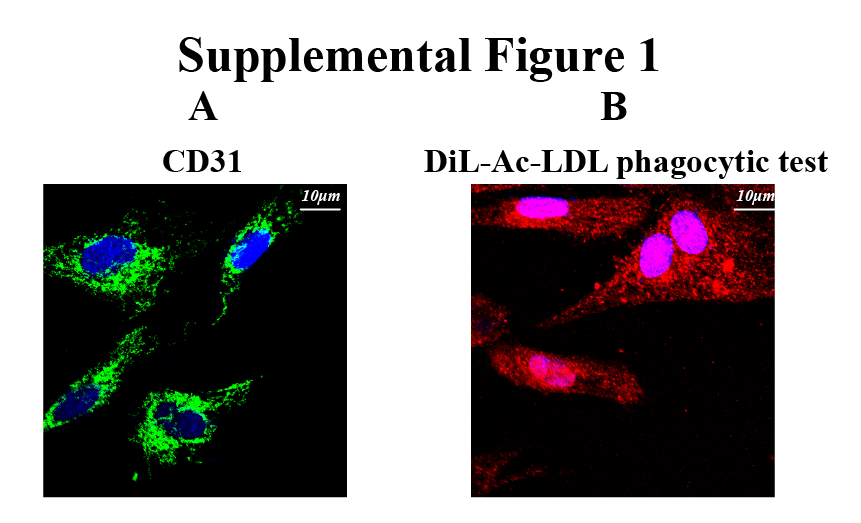
**

**Supplemental Figure 1** **A** CD31 immunocytochemistry of CMECs. **B** Dil-acetylated low-density lipoprotein intake assay. green, CD31; red, Dil-ldl.


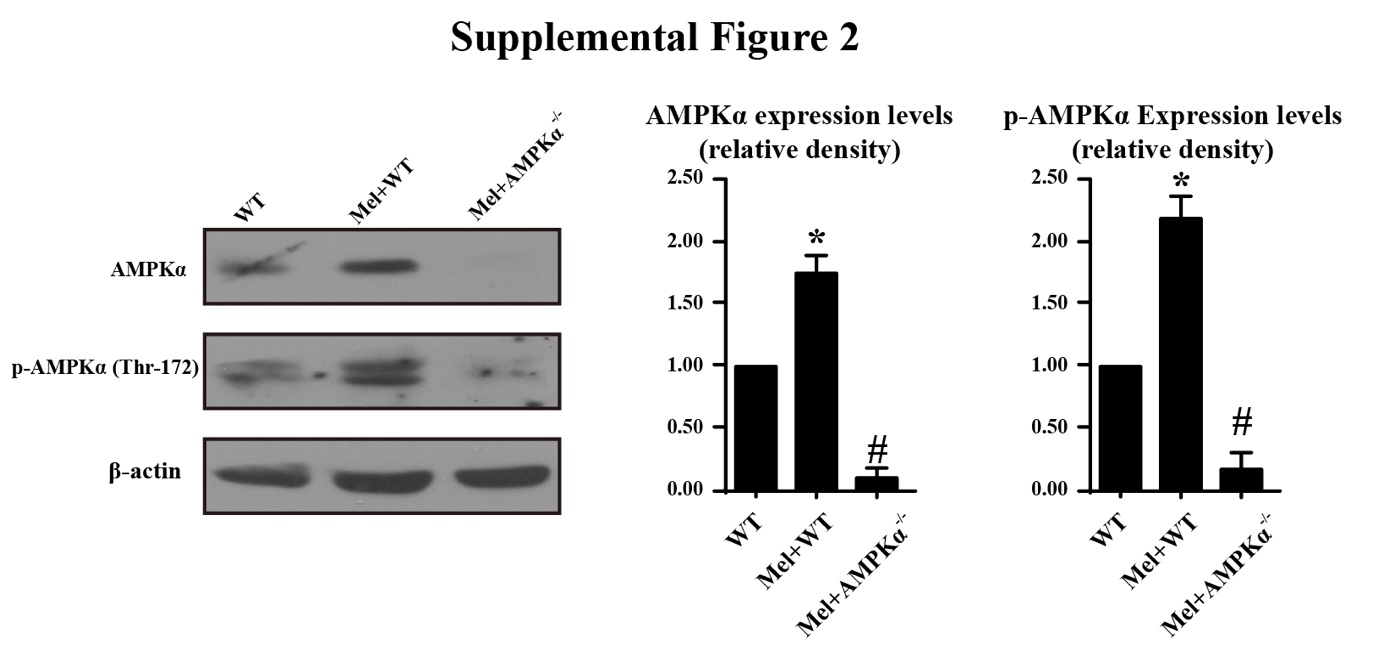


**Supplemental Figure 2** The role of melatonin in the activity of AMPKα. Melatonin could activate AMPKα via phosphorylation of AMPKα at Thr-172 *P<0.05 vs. WT group; #P<0.05 vs. Mel+WT group.


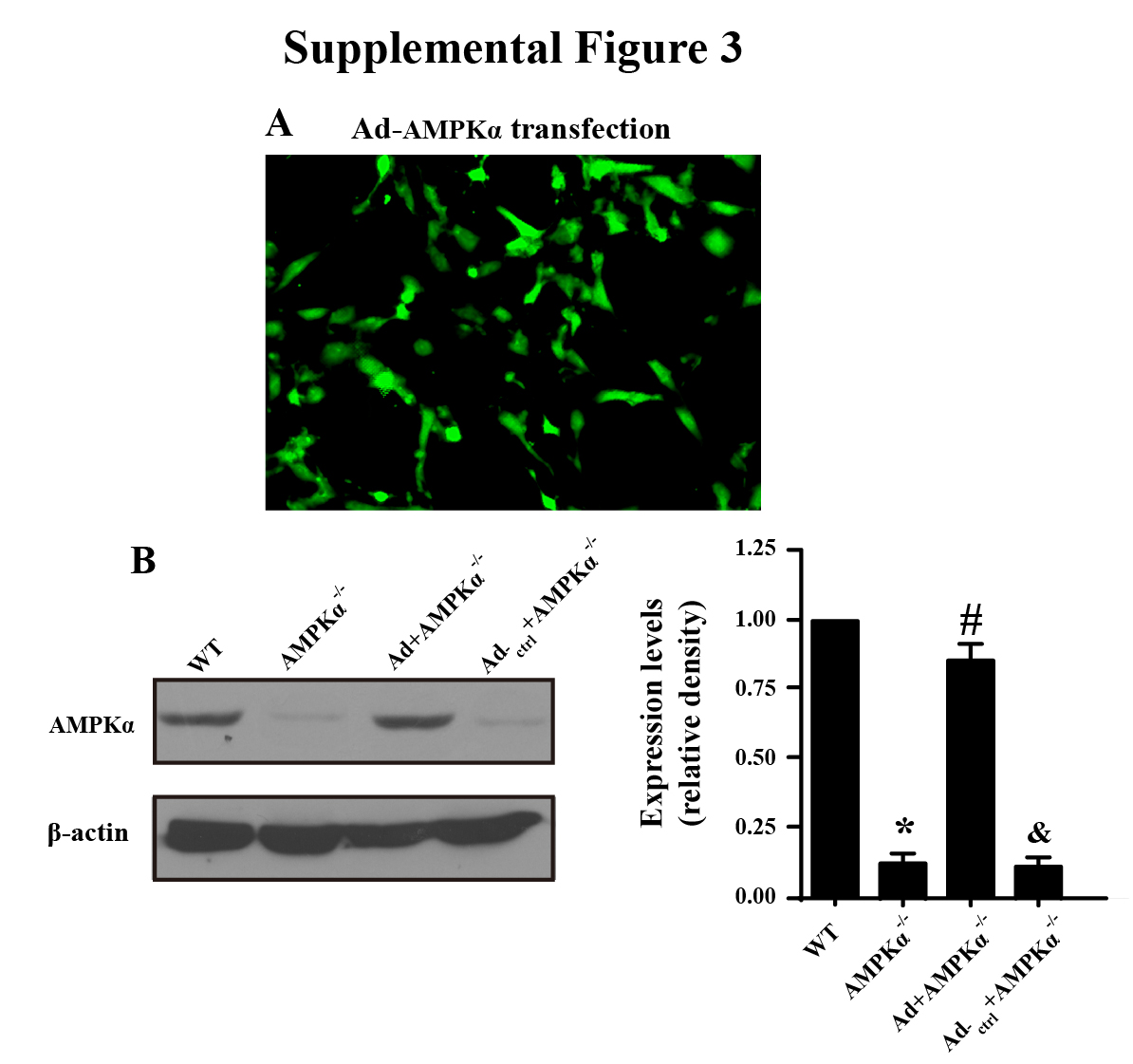


**Supplemental Figure 3 A** The transfection of Ad-AMPKα in CMEC. **B** Western blots were used to assess the AMPKα overexpression by Ad- AMPKα. *P<0.05 vs. WT group; #P<0.05 vs. AMPKα^-/-^ group, &P<0.05 vs. Ad+AMPKα^-/-^ group.
